# Neuroimaging in the Understanding of Acupuncture Analgesia: A Review of Acupuncture Neuroimaging Study Based on Experimental Pain Models

**DOI:** 10.3389/fnins.2021.648305

**Published:** 2021-05-20

**Authors:** Ma Peihong, Qu Yuzhu, Yin Tao, He Zhaoxuan, Cheng Shirui, Teng Yuke, Xie Kunnan, Li Shenghong, Sun Ruirui, Zeng Fang

**Affiliations:** ^1^Acupuncture and Tuina School/The Third Teaching Hospital, Chengdu University of Traditional Chinese Medicine, Chengdu, China; ^2^Acupuncture and Brain Science Research Center, Chengdu University of Traditional Chinese Medicine, Chengdu, China; ^3^State Key Laboratory of Southwestern Chinese Medicine Resources, Innovative Institute of Chinese Medicine and Pharmacy, Chengdu University of Traditional Chinese Medicine, Chengdu, China

**Keywords:** acupuncture, analgesia, neuroimaging, experimental pain model, review

## Abstract

With the development of real-time and visualized neuroimaging techniques, the studies on the central mechanism of acupuncture analgesia gain increasing attention. The experimental pain models have been widely used in acupuncture-analgesia neuroimaging studies with quantitative and controlled advantages. This review aimed to analyze the study design and main findings of acupuncture neuroimaging studies to provide reference for future study. The original studies were collected and screened in English databases (PubMed, EMBASE, and Cochrane Library) and Chinese databases (Chinese Nation Knowledge Infrastructure, Chinese Biomedical Literature Database, the Chongqing VIP Database, and Wanfang Database). As a result, a total of 27 articles were included. Heat stimulation and electroacupuncture were the mostly used pain modeling method and acupuncture modality, respectively. The neuroimaging scanning process can be divided into two models and five subtypes. The anterior cingulate cortex and insula were the most commonly reported brain regions involved in acupuncture analgesia with experimental pain models.

## Introduction

Acupuncture has been used for alleviating pain in China and other oriental countries for thousands of years. It has been accepted as an alternative or complementary therapy for pain in western countries. Nearly half of the 64 recommended indications of acupuncture by WHO (World Health Organization [WHO], 2002 are pain-related disorders.

As the effect of acupuncture analgesia is widely recognized, exploring the mechanism of acupuncture analgesia is always the research hotspot. In the past decades, with the development of real-time and visualized neuroimaging techniques, exploring the central mechanism of acupuncture analgesia had attracted increasing attention. From the 1980s, approximately 90 neuroimaging studies about acupuncture analgesia were published among these studies, about 30% of studies were performed with the experimental pain model. Using the experimental pain model on healthy participants, the researcher can design temporary stimulation quantitatively, and provide quantitative measures of the responses, and the minimized influencing factors. The experimental pain model has been used for exploring the cerebral mechanisms of acupuncture analgesia. It is worth discussing the situation of the experimental pattern for exploring the cerebral response to acupuncture stimulation, and whether the results of the cerebral responses were consistent with the results of clinical pain or not.

Therefore, this review aimed to analyze the pain modeling methods, the acupuncture interventions, neuroimaging techniques, and the cerebral responses to acupuncture stimulation of the acupuncture neuroimaging study with the experimental pain model.

## Methods

### Searching Strategy

Studies were collected by searching the bibliographic database including PubMed, EMBASE, Cochrane Library, Chinese National Knowledge Infrastructure (CNKI), Chinese Biomedical Literature Database (CBM), the Chongqing VIP Database (VIP), and the Wanfang Database (WF). The literature search was conducted from database inception 11th Nov 2020. Details of search terms were modified for each database and depicted in Supplementary Table 1.

The article was included if (1) it was an original article, (2) experimental pain stimulation was conducted on participants, (3) participants received acupuncture as intervention, and (4) the study was performed with neuroimaging techniques (Supplementary Table 1). Articles not fulfilling each of the aforementioned criteria were excluded.

The process of the data selection is described in Supplementary Figure 2.

### Data Extraction and Analysis

Information including the year of publication, the corresponding author (name, institution), the trial place, the method of modeling, the acupuncture intervention (modality, the selection of acupoints), the pain assessment, the neuroimaging techniques, and the results of brain regions involved in acupuncture analgesia were extracted.

## Results

A total of 27 neuroimaging studies with experimental pain models performing on healthy participants were included in this review.

### The Basic Information of the Studies

The first study was published in 1980 in Chinese. Sixteen of these 27 studies were conducted in China, and 9 studies in the United States. There were 13 corresponding affiliations mentioned; the top two were Massachusetts General Hospital (6 studies) and Peking University (4 studies).

### The Pain Modeling Method

**FIGURE 1:**
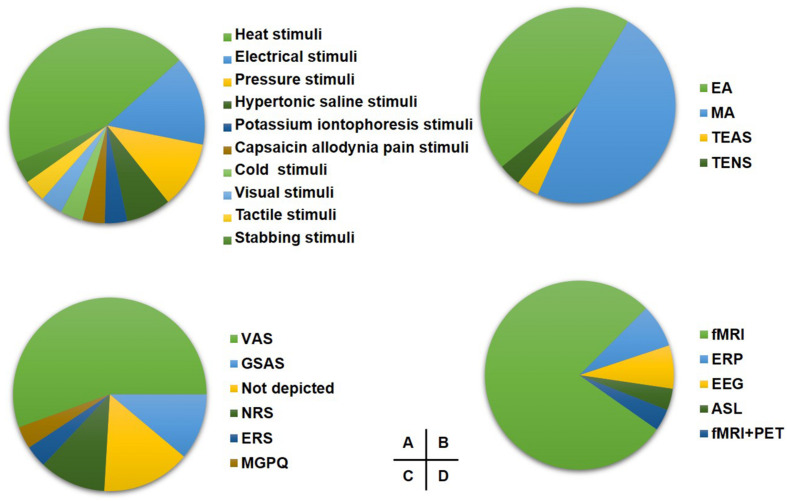
The study design of included studies. **(A)** The proportion of induced pain modality. **(B)** The proportion of acupuncture modality for experimental pain. **(C)** The proportion of pain assessment scale. **(D)** The proportion of imaging techniques. EA, electroacupuncture; MA, manual acupuncture; TEAS, transcutaneous electrical nerve stimulation; TENS, transcutaneous electrical nerve; VAS, visual analog scale; GSAS, gracely sensory and affective scales; NRS, numerical rating scale; ERS, expectations for relief scale; MGPQ, McGill pain questionnaire; fMRI, functional MRI; ERP, event-related potential.

The pain modeling methods included heat stimulation (11 studies), electrical stimulation (4 studies), pressure stimulation (3 studies), injections of hypertonic saline (2 studies), potassium iontophoresis stimulation (2 studies), cold pain stimulation (1 study), video stimulation (1 study), tactile stimulation (1 study), stabbing stimulation (1 study), and capsaicin allodynia stimulation (1 study) on healthy participants (Figure 1A).

### Intervention Protocol

#### Acupuncture Modality

Thirteen studies chose manual acupuncture (MA), and 12 studies chose electroacupuncture (EA) as the intervention method. Besides, the transcutaneous electrical nerve (TENS) and transcutaneous electric acupoint stimulation (TEAS) were applied in one study, respectively (Figure 1B).

#### Acupoint Selection

Hegu (LI 4) (9 studies), Zusanli (ST 36) (8 studies), and Sanyinjiao (SP 6) (4 studies) were the most frequently used acupoints in these studies (Supplementary Table 4).

#### Pain Assessment

Fifteen studies assessed the pain with the Visual Analogical Scale (VAS), and three studies applied the Gracely Sensory and Affective Scales (GSAS). Other pain assessment scales are shown in Figure 1C.

### Scanning Methods

#### Scanning Techniques

Twenty-one studies applied fMRI, two studies used event-related potentials (ERP), two studies used electroencephalogram (EEG), one study used arterial spin labeling (ASL), and one study used both fMRI and PET as neuroimaging techniques (Figure 1D).

#### Scanning Imaging With Process

**FIGURE 2:**
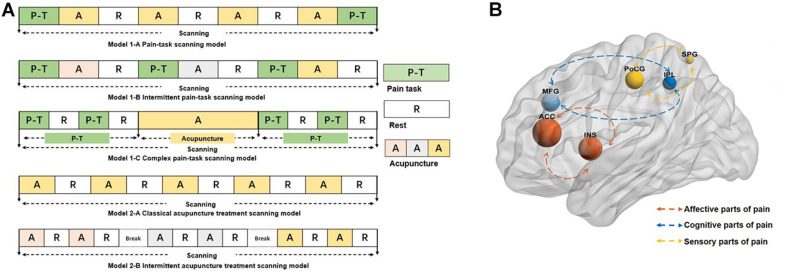
**(A)** The models of neuroimaging scanning. The different color A represents the different intensity or modality of acupuncture. **(B)** The reported brain regions in the studies. The size of the nodes represents the frequency the reported brain regions, the different color of the nodes represents the different roles of the process of the pain. The blue nodes mainly represent the role of processing the cognitive parts of pain, the red nodes mainly represent the role of processing the affective parts of pain, and the yellow nodes mainly represent the role of processing the sensory part of pain. ACC, anterior cingulate cortex; INS, insula; MFG, middle frontal gyrus; PoCG, postcentral gyrus; IPL, inferior parietal gyrus; SPG, superior parietal gyrus.

Similar scanning processes were sorted together regardless of the scanning time and repetition times. Scanning processes can be divided into model 1 and model 2 based on whether the pain stimulation was a part of task scanning. Subsequently, according to the sequence of pain stimulation, model 1 was divided into three subtypes [model 1-A (Kong et al., 2006, 2009a,b; Zyloney et al., 2010; Shi et al., 2015; Peng et al., 2019, model 1-B (Lin et al., 2004; Shukla et al., 2011; Leung et al., 2014; Lee et al., 2015, model 1-C (Zhang et al., 2003b; Theysohn et al., 2014; Niu et al., 2017], and according to the breaks between acupuncture treatment, model 2 was divided into two subtypes [model 2-A (Research of Acupuncture Anesthesia in Beijing, 1980; Zhang et al., 2003a; Jiang et al., 2014; Lin et al., 2018 and model 2-B (Zeng et al., 2006; Cheng et al., 2007; Dougherty et al., 2008; Ziping et al., 2013; Cao et al., 2019] (Figure 2A).

### Cerebral Responses to Acupuncture Stimulation

The reported deactivated/activated brain regions are shown in Supplementary Table 4. The high-frequency reported brain regions (regardless of the increased or decreased regions) are shown in Figure 2B. ACC was the most frequent brain region involving in the acupuncture analgesia studies.

## Discussion

This review mainly described the current status of the central mechanism research of acupuncture analgesia based on the experimental pain model and neuroimaging techniques.

### The Pain Modeling

Since early 1980, researchers have applied the electrical experimental pain model to investigate the central mechanism of acupuncture analgesia based on the ERP technique (Research of Acupuncture Anesthesia in Beijing, 1980. Compared with the patients with pain, the experimental pain model on healthy participants can control the characteristics of the pain including localization, time, frequency, and modality, and provide the quantitative assessment for the evoked response [psychophysical, neurophysiological, and biochemical markers (Drewes et al., 2003; Staahl et al., 2006, 2009; Olesen et al., 2009] and minimize the confounding factors in the diseased condition.

Among the 27 articles in this review, the most common pain modeling method was heat stimulation (Figure 1A). The reproducibility of heat stimulation without provoking changes in cutaneous sensitivity and easy implementation with neuroimaging techniques may be the reasons for its frequent application (Olesen et al., 2009. In addition, electrical pain stimuli, and pressure stimuli were also the high-frequency pain modeling methods in this review; these methods mainly induced neuropathic pain on the skin. However, except for the neuropathic pain mostly applied in the skin to explore the central mechanisms of acupuncture analgesia, the muscle and visceral pain using specific stimulations deserve more attention to broaden the recognition of acupuncture analgesia in the experimental pain.

### The Design of Acupuncture Intervention

In these 27 studies, the application of MA accounted for 48% and EA accounted for 44%. MA was chosen to explore the underlying central mechanism of other influencing factors such as the placebo effect (vs. Streitberger needle) (Streitberger and Kleinhenz, 1998; Dougherty et al., 2008; Kong et al., 2009b; Zyloney et al., 2010; Niu et al., 2017; Cao et al., 2019 or acupoint specification (Kong et al., 2006, 2009a; Zeng et al., 2006; Leung et al., 2014 of acupuncture analgesia. MA was adopted by most neuroimaging studies as the intervention method. Despite the wide application of MA, the stimulation of MA is hard to be quantified for the individual differences of manipulation induced by different practitioners (Qiu et al., 2016. To ensure the consistency and reproducibility of the trial, researchers should make the explicit protocol of the acupuncture manipulation in detail and employ the same acupuncturist when designing the trial. When using EA, researchers usually explored the mechanisms of the different intensities of EA (2 vs. 100 Hz) for acupuncture analgesia. The stimulation produced by EA has the advantages of controlling and high reproducibility, which has wide application in the research field. However, researchers mainly focused on the frequencies of EA and paid little attention to the intensities, time, and waveform of EA. Therefore, exploring other influencing factors such as intensities, time, and waveform of EA can fully understand the central mechanism of acupuncture analgesia on experimental pain.

Hegu (LI 4) (9 studies) (Zeng et al., 2006; Dougherty et al., 2008; Kong et al., 2009a,b; Zyloney et al., 2010; Jiang et al., 2014; Theysohn et al., 2014; Lee et al., 2015; Niu et al., 2017, Zusanli (ST 36) (8 studies) (Weiting et al., 2003; Zhang et al., 2003a,b; LaMei et al., 2013a,b; Theysohn et al., 2014; Lin et al., 2018; Wenjin et al., 2020, and Sanyinjiao (SP 6) (4 studies) (Weiting et al., 2003; Zhang et al., 2003a,b; Cao et al., 2019 (Supplementary Table 4) were the most used acupoint selection in these 27 studies. The aforementioned acupoints were widely used for pain relief in the clinic (Zeng et al., 2009; Fofi et al., 2014; Wang et al., 2015. Besides, the acupoints were located on the four limbs which were commonly used because they were easy to perform acupuncture manipulation during the scanning.

This review showed that VAS is the preferred scale for assessing pain. Using VAS was previously recommended in both research and clinical practice with the advantages of its simplicity and adaptability (Renskers et al., 2018. Although researchers mostly rely on the visual and simple numeric forms to assess the pain, the single assessment cannot reflect the pain. Pain is a multidimensional subjective experience (Wideman et al., 2019; the assessment can be done from a multidimensional and multimodal approach, for example, researchers can assess the pain qualitatively (words and behaviors) (Prkachin, 2009; Wideman et al., 2018 and quantitatively (self-reported measures and non-self-reported measures) (Backonja et al., 2013; Wager et al., 2013 to comprehensively reflect the pain perception.

### The Application of Neuroimaging Techniques

Among the neuroimaging techniques, fMRI (85%) was the most used technique of these 27 studies. fMRI indirectly measures brain activity by detecting associated changes in blood flow (hemodynamic response) (Matthews and Jezzard, 2004. It is an extremely useful measurement in acute and experimental pain where there are short periods of pain followed by short periods that are pain-free, causing a rapidly changing hemodynamic response (Morton et al., 2016. Different from the clinical pain by the resting-state scanning (Desouza et al., 2013; Chen et al., 2014; Li et al., 2016, the acupuncture for experimental pain generally applied the task neuroimaging scanning to detect the brain activities. Also, the block design of task-fMRI is the most used experimental scanning paradigm. The models of the scanning process are shown in Figure 2A. Each model has its characteristics.

The neuroimaging processes can be divided into model 1 and model 2 according to whether the pain stimulation was performed during scanning. Model 1 can be classified into three subtypes according to the sequence of pain stimulation. For model 1-A, it is marked that the pain stimulation existed pre- and post-acupuncture. Setting the identical pain stimulation can be applied to evaluate the efficacy of acupuncture analgesia while setting it intentionally different could be applied to explore the expectancy of the acupuncture analgesia (Kong et al., 2006, 2009a,b. The characteristic of model 1-B is that each acupuncture session is performed by different modalities or intensities. This model is used to explore mechanisms of the influencing factors for acupuncture analgesia. Also, for model 1-C, it is characterized that the pain stimulation is composed of several blocks, which can explore the dynamic alterations from baseline to post-acupuncture. Differing from that of model 1, there was no pain task in model 2. Model 2-A is characterized by the classical block acupuncture design “rest-stimulation-rest-stimulation.” Also, model 2-B is that the acupuncture session is performed for several on–off scans with breaks. The several different on–off scans could explore the multiple factors such as the intensity or others that influenced the acupuncture analgesic effect.

During the experiment, the activated brain region of the immediate efficacy and post-efficacy of acupuncture may overlap (Qin et al., 2008; therefore, when designing the trial to explore the acupuncture analgesia by task neuroimaging scanning on experimental trial, researchers can extend the resting period between the scanning of acupuncture stimulation to decrease the post-efficacy of acupuncture to the minimum.

### The Cerebral Response to Acupuncture Stimulation

Pain is a multidimensional subjective experience generally including the sensory (intensity, location), affective (unpleasantness, fear), and cognitive factors (memory, attention) (Schnitzler and Ploner, 2000. The signal processing of pain in the brain is highly interconnected (Coghill et al., 1999. Of the core brain regions of processing pain, ACC and insula participate in the coding of pain unpleasantness and other emotional sensations (Rainville et al., 1997; Kulkarni et al., 2005; Gasquoine, 2014. The primary somatosensory cortex (SI), the second somatosensory cortex (SII), and insula are mainly involved in the sensory-discriminative aspect of pain such as location, intensity, and so on (Bushnell et al., 1999; Coghill et al., 1999; Brooks et al., 2005; in this review, postcentral gyrus (PoCG) and superior parietal gyrus (SPG) belonging to SI/SII parts are frequently activated regions. In addition, the parietal lobe and prefrontal cortex participate in the cognition of pain such as memory and attention of pain (Coghill et al., 1999; Cannon et al., 2005. The inferior parietal gyrus (IPL) and middle frontal gyrus (MFG) were the activated brain regions with high frequency of these included articles.

In this review, ACC and insula were the most commonly reported brain regions in the experimental pain for acupuncture analgesia (Figure 2B). From other studies about acupuncture for chronic clinical pain, the brain response to acupuncture stimuli also encompasses a broad network of regions including somatosensory, affective, and cognitive processing (Huang et al., 2012; Villarreal Santiago et al., 2016; Li et al., 2017. Analysis of experimental pain neuroimaging for acupuncture analgesia shows consistently activated regions with the results of clinical pain. The consistency of the activated regions in the acupuncture for chronic and acute pain indicated that experimental pain was a valuable complementary and alternative method to explore the central mechanism of acupuncture analgesia with the ignored advantages of simplicity, controlled parameters, and reproducibility.

The complex brain networks participated in the central processing of acupuncture analgesia for experimental pain (Morton et al., 2016. The reported brain regions of this review mainly focused on the processing of the sensory and affective parts (ACC and insula) (Rainville et al., 1997; Gasquoine, 2014 in the experimental pain. It reflected the participation of the sensory and emotional parts of the pain processed the acupuncture analgesia. Therefore, give enough consideration to the influence of the sensory and emotions during the trial, and make an explicit protocol to guarantee the consistency of implementation and decrease the heterogeneity of the results.

In conclusion, this review showed the situation of acupuncture analgesia studies with experimental pain and neuroimaging techniques. From this review, some suggestions can be provided for future study design. (1) The selection of the types of experimental pain could be broadened but not limited to the neuropathic pain on the skin. (2) Researchers can evaluate the pain from multimodal dimensions and using more objective assessment methods to guarantee the objective results and profoundly shape the experience of pain. (3) Researchers can use the neuroimaging technologies to compare the central mechanisms of EA and MA at different acupoints, different stimulations, and different intensities, to give the reference for the selection of the clinical treatment.

## Author Contributions

ZF and LS designed the study. MP, CS, and QY participated in screening studies. YT and XK extracted the data from the included studies. HZ and CS performed the analysis plan. MP, SR, and YT drafted the article. ZF revised the draft. All authors have read and approved the publication of the final article.

## Conflict of Interest

The authors declare that the research was conducted in the absence of any commercial or financial relationships that could be construed as a potential conflict of interest.

## Abbreviations

CNKI: Chinese National Knowledge Infrastructure
CBM: Chinese Biomedical Literature Database
VIP: the Chongqing VIP Database
WF: the Wanfang Database
EA: electroacupuncture
MA: manual acupuncture
TENS: transcutaneous electrical nerve
TEAS: transcutaneous electric acupoint stimulation
VA: Visual Analogue Scale
GSAS: Gracely Sensory and Affective Scale
fMRI: functional magnetic resonance imaging
ERP: event-related potential
EEG: electroencephalogram
ACC: anterior cingulate cortex
MFG: medial frontal gyrus
SI: primary somatosensory cortex
SII: second somatosensory cortex
PoCG: postcentral gyrus
SPG: superior parietal gyrus
IPL: inferior parietal gyrus.
