# Supplementary figures and images for: Neuroimaging in the Understanding of Acupuncture Analgesia: A Review of Acupuncture Neuroimaging Study Based on Experimental Pain Models
Source: Front Neurosci. 2021 May 20;15:648305. doi: 10.3389/fnins.2021.648305 (PMC8172961; doi:10.3389/fnins.2021.648305)

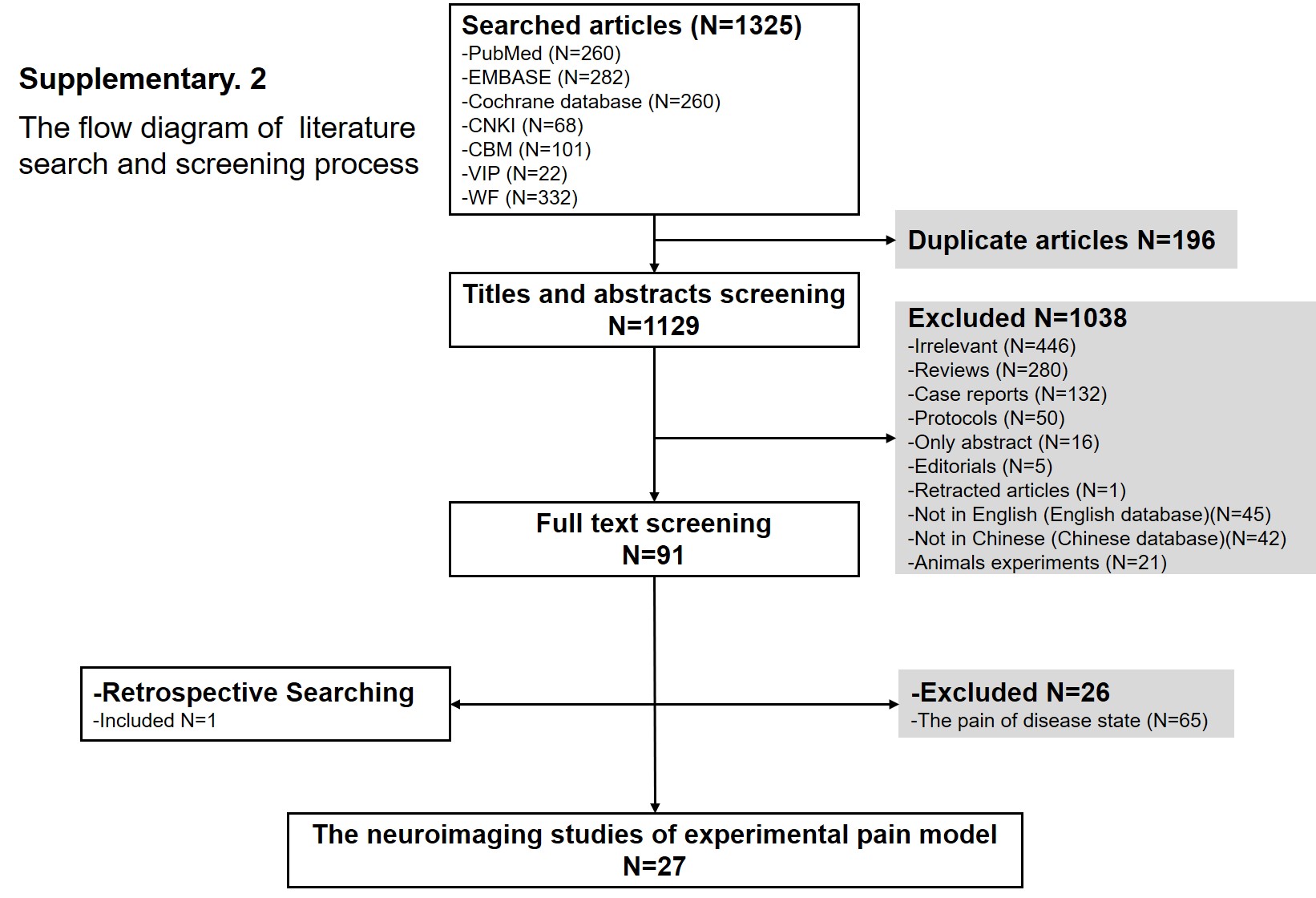

Supplement: Supplementary file 1 [file Image_1.JPEG]
