# Supplementary material for: Neuroimaging in the Understanding of Acupuncture Analgesia: A Review of Acupuncture Neuroimaging Study Based on Experimental Pain Models
Source: Front Neurosci. 2021 May 20;15:648305. doi: 10.3389/fnins.2021.648305 (PMC8172961; doi:10.3389/fnins.2021.648305)
Supplement: Supplementary file 2 [file Table_1.DOCX]

| **Supplementary Table 1. Full search strategy for each of the electronic databases queried.** | | | |
| --- | --- | --- | --- |
| **Databases** | **Coverage** | **Searches** | **Hits** |
| PubMed Database | Date of inception  – Nov 11th, 2020 | 1. (((((((neuroimaging[MeSH Terms]) OR (neuroimaging[All fields])) OR ((neuroimaging[MeSH Terms]) OR (neuroimaging[All fields]))) OR (("magnetic resonance imaging"[MeSH Terms]) AND ("magnetic resonance imaging"))) OR ((Positron-Emission Tomography[MeSH Terms]) AND (Positron-Emission Tomography))) OR ((functional magnetic resonance imaging) OR (fMRI))) OR ((Diffusion Tensor Imaging) OR (DTI))) OR ((structural magnetic resonance imaging) OR (sMRI))  2. ((pain[MeSH Terms]) AND (pain)) OR ((Analgesia[MeSH Terms]) AND (Analgesia))  3. ((((((((((Acupuncture Therapy[MeSH Terms]) OR (acupuncture[MeSH Terms])) OR (Acupuncture Analgesia[MeSH Terms]))) OR (Acupuncture Therapy)) OR (Acupuncture)) OR (Acupuncture Points)) OR (acupressure)) OR (electroacupuncture)) OR (meridians)) OR (moxibustion)  4. #1 AND #2 AND #3 | 260 |
| EMBASE Database | Date of inception  -Nov 11th, 2020 | #1. 'acupressure'/exp OR acupressure OR 'electroacupuncture'/exp OR electroacupuncture OR 'meridians'/exp OR meridians OR 'moxibustion'/exp OR moxibustion  #2. 'acupuncture therapy'/exp OR 'acupuncture therapy' OR 'acupuncture'/exp OR 'acupuncture' OR 'acupuncture points'/exp OR 'acupuncture points'  #3. acupoint$  #4. meridian$  #5. 'deqi'/exp OR deqi  #6. moxibustion$  #7. #1 OR #2 OR #3 OR #4 OR #5 OR #6  #8. 'pain'/exp OR pain  #9. 'analgesia'/exp OR analgesia  #10. #7 AND #8 AND #9  #11. 'neuroimaging'/exp OR neuroimaging OR 'magnetic resonance imaging'/exp OR 'magnetic resonance imaging' OR 'mri'/exp OR mri OR 'structural magnetic resonance imaging'/exp OR 'structural magnetic resonance imaging' OR smri OR 'diffusion tensor imaging'/exp OR 'diffusion tensor imaging' OR 'dti' OR 'functional magnetic resonance imaging'/exp OR 'functional magnetic resonance imaging' OR 'fmri'/exp OR 'fmri' OR 'positron emission tomograph' OR 'pet'/exp OR pet  #12. #10 AND #11 | 282 |
| Cochrane database | Date of inception  – Nov 11th, 2020 | #1 “Acupuncture Therapy” or “Acupuncture” or “Acupuncture Points”  #2 acupressure or electroacupuncture or meridians or moxibustion  #3 acupoint$  #4 meridian$  #5 deqi  #6 moxibustion$  #7 pain  #8 analgesia  #9 neuroimaging OR ‘magnetic resonance imaging' OR mri OR 'structural magnetic resonance imaging' OR smri OR 'Diffusion Tensor Imaging' OR 'DTI' OR ‘functional magnetic resonance imaging’ OR ‘fmri’ OR 'positron emission tomograph' OR pet  #10 #1 OR #2 OR #3 OR #4 OR #5 OR #6  #11 #7 OR #8  #12 #10 AND #11  #13 #9 AND #12 | 260 |
| Chinese Nation Knowledge Infrastructure  (CNKI, Chinese Database) | Date of inception  – Nov 11th, 2020 | (((((SU = 针灸) OR (SU = 针刺)) OR (SU = 电针)) OR (SU = 针法)) AND (((SU = 疼痛) OR (SU = 痛)) OR (SU = 镇痛))) AND (((((((SU = 神经影像) OR (SU = 脑影像)) OR (SU = 磁共振技术)) OR (SU = 功能磁共振)) OR (SU = 结构磁共振)) OR (SU = 弥散张量成像)) OR (SU = PET))) [Journal article] | 68 |
| Chinese Biomedical Literature Database  (CBM, Chinese Database) | Date of inception  – Nov 11th, 2020 | (("针刺"[核心字段:智能] OR "针灸"[核心字段:智能] OR "电针"[核心字段:智能] OR "针法"[核心字段:智能]) AND ( "痛"[核心字段:智能] OR "疼痛"[核心字段:智能] OR "镇痛"[核心字段:智能] )) AND ("神经影像"[核心字段:智能] OR "脑成像"[核心字段:智能] OR "磁共振技术"[核心字段:智能] OR "功能磁共振"[核心字段:智能] OR "结构磁共振"[核心字段:智能] OR "弥散张量成像"[核心字段:智能] OR "PET"[核心字段:智能]) | 101 |
| Chongqing VIP Database  (VIP, Chinese Database) | Date of inception  – Nov 11th, 2020 | ((K=疼痛 OR K=痛) OR K=镇痛) AND (((((K=神经影像 OR K=脑影像) OR K=磁共振技术) OR K=功能磁共振) OR K=结构磁共振) OR K=PET) AND (((K=针刺 OR K=针法) OR K=电针) OR K=针灸) | 22 |
| Wanfang Database  (WF, Chinese Database ) | Date of inception  – Nov 11th, 2020 | ((针刺 or 针灸 or 针法 or 电针) and (痛 or 疼痛 or 镇痛) and (神经影像 or 脑影像 or 磁共振技术 or 功能磁共振 or 结构磁共振 or 弥散张量成像 or PET)) | 332 |

**Neuroimaging methods performed by the following scanning techniques can be included:**

[Diffusion Tensor Imaging (DTI), functional Magnetic Resonance Imaging (fMRI), Magnetic Resonance Spectroscopy (MRS), Arterial Spin Labeling (ASL), Positron Emission Tomography (PET), Single Photon Emission Computed Tomography (SPECT), Electroencephalogram (EEG), Event-Related Potentials (ERP)]
