# Supplementary material for: Neuroimaging in the Understanding of Acupuncture Analgesia: A Review of Acupuncture Neuroimaging Study Based on Experimental Pain Models
Source: Front Neurosci. 2021 May 20;15:648305. doi: 10.3389/fnins.2021.648305 (PMC8172961; doi:10.3389/fnins.2021.648305)
Supplement: Supplementary file 3 [file Table_2.DOCX]

**Supplementary Table 2. The basic information of the 27 included studies.**

| Study  No. | Title | Corresponding Author | Institution | Perform Place | Publish Time | Journal | Language |
| --- | --- | --- | --- | --- | --- | --- | --- |
| S01(1) | Neurobiological mechanisms of TENS-induced analgesia | L. Hu | Chinese Academy of Sciences | CHN | 2019 | Neuroimage | ENG |
| S02(2) | Analgesic Effects Evoked by Real and Imagined Acupuncture: A Neuroimaging Study | Jian Kong | Massachusetts General Hospital | USA | 2018 | Cerebral cortex | ENG |
| S03(3) | When pain is not only pain: inserting needles into the body evokes distinct reward-related brain responses in the context of a treatment | Younbyoung Chae | Kyung Hee University | KOREA | 2015 | Physiology & behavior | ENG |
| S04(4) | The effect of acupuncture needle combination on central pain processing--an fMRI study | Albert Leung | The University of California | USA | 2014 | Molecular pain | ENG |
| S05(5) | The analgesic effect of electroacupuncture on acute thermal pain perception--a central neural correlate study with fMRI | Albert Leung | The University of California | USA | 2011 | Molecular pain | ENG |
| S06(6) | Imaging the functional connectivity of the Periaqueductal Gray during genuine and sham electroacupuncture treatment | Jian Kong | Massachusetts General Hospital | USA | 2010 | Molecular pain | ENG |
| S07(7) | Expectancy and treatment interactions: a dissociation between acupuncture analgesia and expectancy evoked placebo analgesia | Jian Kong | Massachusetts General Hospital | USA | 2009 | Neuroimage | ENG |
| S08(8) | An fMRI study on the interaction and dissociation between expectation of pain relief and acupuncture treatment | Jian Kong | Massachusetts General Hospital | USA | 2008 | Neuroimage | ENG |
| S09(9) | A combined [11C] diprenorphine PET study and fMRI study of acupuncture analgesia | Darin D. Dougherty | Massachusetts General Hospital | USA | 2008 | Behavioral brain research | ENG |
| S10(10) | Brain activity associated with expectancy-enhanced placebo analgesia as measured by functional magnetic resonance imaging | Jian Kong | Massachusetts General Hospital | USA | 2006 | The Journal of Neuroscience | ENG |
| S11(11) | Relations between brain network activation and analgesic effect induced by low vs. high frequency electrical acupoint stimulation in different subjects: a functional magnetic resonance imaging study | JiSheng Han | Peking University | CHN | 2003 | Brain research | ENG |
| S12(12) | Acupuncture-related modulation of pain-associated brain networks during electrical pain stimulation: a functional magnetic resonance imaging study | Nina Theysohn | University Hospital Essen | GERMANY | 2014 | The journal of alternative and complementary medicine | ENG |
| S13(13) | Modulation of pain signal processing by electric acupoint stimulation: an electroencephalogram study (Chinese version) | Jisheng Han | Peking University | CHN | 2003 | Journal of Peking University | CN |
| S14(14) | Electroacupuncture modulates cortical activities evoked by noxious somatosensory stimulations in human | Jing Shi | Huazhong University of Science and Technology | CHN | 2006 | Brain research | ENG |
| S15(15) | Event-related potential evoked by suggestive sensitivity and acupuncture on pain stimuli (Chinese version) | Research of acupuncture anesthesia in Beijing | Research of acupuncture anesthesia in Beijing | CHN | 1980 | Journal of Psychology | CN |
| S16(16) | Brain Network Response to Acupuncture Stimuli in Experimental Acute Low Back Pain: An fMRI Study | Wen Wu | Southern Medical University | CHN | 2015 | Evidence-Based Complementary and Alternative Medicine | ENG |
| S17(17) | Pain matrix response to acupuncture stimuli in individuals with acute Low back pain: an fmri study(Chinese version) | Wen Wu | Southern Medical University | CHN | 2013 | Chinese Journal of Pain Medicine | CN |
| S18(18) | Modulation of cold pain in human brain by electric acupoint stimulation: evidence from Fmri | Fei Luo | Peking University | CHN | 2003 | Neuroreport | ENG |
| S19(19) | Cerebral blood flow-based evidence for mechanisms of low- versus high-frequency transcutaneous electric acupoint stimulation analgesia: a perfusion fMRI study in humans | C.Cui | Peking University | CHN | 2014 | Neuroscience | ENG |
| S20(20) | Investigation of analgesic mechanism of acupuncture: a fMRI study(Chinese version) | Yuanfeng Li | Beijing Tiantan Hospital | CHN | 2004 | Chinese Journal of Medical Imaging Technology | CN |
| S21(21) | Expertise modulates the perception of pain in others | Jean Decety | The University of Chicago | USA | 2007 | Current biology | ENG |
| S22(22) | Effect of Acupuncture Stimulation of Zusanli (ST 36) on Cerebral Regional Homogeneity in Volunteer Subjects with Different Constitutions: A Resting state fMRI Study | Xuezhi Li | Chongqing Medical University | CHN | 2013 | Acupuncture Research | CN |
| S23(23) | Acupuncture analgesia effect in different sensitive constitution: a resting-state fMRI study | Xuezhi Li | Chongqing Medical University | CHN | 2013 | Journal of The Third Military Medicine University | CN |
| S24(24) | ASL-based Observation of Central Nervous System Responses to Acupuncture Analgesia for People with Different Sensitivities (Chinese version) | Xuezhi Li | ChongQing Medical University | CHN | 2018 | Acupuncture Research | CN |
| S25(25) | Interaction of acupuncture treatment and manipulation laterality modulated by the default mode network | Jie Tian | Chinese Academy of Sciences | CHN | 2017 | Molecular pain | ENG |
| S26(26) | Deqi and Sharp pain during acupuncture at Taichong eliciting the opposite functional brain network effects- an fMRI study | Jiliang Fang | Chinese Academy of Sciences | CHN | 2012 | Chinese imaging journal of integrated traditional and western medicine | CN |
| S27(27) | A preliminary study on the central analgesic mechanism of acupuncturing the stomach meridian foot-Yangming by fMRI | Junping Zhen | Shanxi Medical University | CHN | 2020 | Chinese Journal Magnetic Resonance Imaging | CN |

**Reference**

1. Peng WW, Tang ZY, Zhang FR, Li H, Kong YZ, Iannetti GD, et al. Neurobiological mechanisms of TENS-induced analgesia. Neuroimage. 2019;195:396-408.

2. Cao J, Tu Y, Orr SP, Lang C, Park J, Vangel M, et al. Analgesic Effects Evoked by Real and Imagined Acupuncture: A Neuroimaging Study. Cereb Cortex. 2019;29(8):3220-31.

3. Lee IS, Wallraven C, Kong J, Chang DS, Lee H, Park HJ, et al. When pain is not only pain: inserting needles into the body evokes distinct reward-related brain responses in the context of a treatment. Physiology & behavior. 2015;140:148-55.

4. Leung A, Zhao Y, Shukla S. The effect of acupuncture needle combination on central pain processing--an fMRI study. Molecular pain. 2014;10:23.

5. Shukla S, Torossian A, Duann JR, Leung A. The analgesic effect of electroacupuncture on acute thermal pain perception--a central neural correlate study with fMRI. Molecular pain. 2011;7:45.

6. Zyloney CE, Jensen K, Polich G, Loiotile RE, Cheetham A, LaViolette PS, et al. Imaging the functional connectivity of the Periaqueductal Gray during genuine and sham electroacupuncture treatment. Molecular pain. 2010;6:80.

7. Kong J, Kaptchuk TJ, Polich G, Kirsch I, Vangel M, Zyloney C, et al. Expectancy and treatment interactions: a dissociation between acupuncture analgesia and expectancy evoked placebo analgesia. Neuroimage. 2009;45(3):940-9.

8. Kong J, Kaptchuk TJ, Polich G, Kirsch I, Vangel M, Zyloney C, et al. An fMRI study on the interaction and dissociation between expectation of pain relief and acupuncture treatment. Neuroimage. 2009;47(3):1066-76.

9. Dougherty DD, Kong J, Webb M, Bonab AA, Fischman AJ, Gollub RL. A combined [11C]diprenorphine PET study and fMRI study of acupuncture analgesia. Behavioural brain research. 2008;193(1):63-8.

10. Kong J, Gollub RL, Rosman IS, Webb JM, Vangel MG, Kirsch I, et al. Brain activity associated with expectancy-enhanced placebo analgesia as measured by functional magnetic resonance imaging. The Journal of neuroscience : the official journal of the Society for Neuroscience. 2006;26(2):381-8.

11. Zhang WT, Jin Z, Cui GH, Zhang KL, Zhang L, Zeng YW, et al. Relations between brain network activation and analgesic effect induced by low vs. high frequency electrical acupoint stimulation in different subjects: a functional magnetic resonance imaging study. Brain research. 2003;982(2):168-78.

12. Theysohn N, Choi KE, Gizewski ER, Wen M, Rampp T, Gasser T, et al. Acupuncture-related modulation of pain-associated brain networks during electrical pain stimulation: a functional magnetic resonance imaging study. Journal of alternative and complementary medicine (New York, NY). 2014;20(12):893-900.

13. Weiting Z, Fei L, Yingwei Q, Ying W, Jingyu Z, J.WOODWORD D, et al. Modulation of pain signal processing by electric acupoint stimulation: an electroencephalogram study (Chinese version). Journal of Peking University. 2003;35(3):236-40.

14. Zeng Y, Liang XC, Dai JP, Wang Y, Yang ZL, Li M, et al. Electroacupuncture modulates cortical activities evoked by noxious somatosensory stimulations in human. Brain research. 2006;1097(1):90-100.

15. Beijing RGoAAi. Event-related potential evoked by suggestive sensitivity and acupuncture on pain stimuli(Chinese version). Journal of Psychology. 1980(2).

16. Shi Y, Liu Z, Zhang S, Li Q, Guo S, Yang J, et al. Brain Network Response to Acupuncture Stimuli in Experimental Acute Low Back Pain: An fMRI Study. Evidence-based Complementary and Alternative Medicine. 2015;2015.

17. Ziping L, Wen W, Shanshan Z, Shigui G, Jianming Y. Pain matrix response to acupuncture stimuli in individuals with acute Low back pain: an fmri study (Chinese version). Chinese Journal of Pain Medicine. 2013;19(4):201-5,.

18. Zhang WT, Jin Z, Huang J, Zhang L, Zeng YW, Luo F, et al. Modulation of cold pain in human brain by electric acupoint stimulation: evidence from fMRI. Neuroreport. 2003;14(12):1591-6.

19. Jiang Y, Liu J, Liu J, Han J, Wang X, Cui C. Cerebral blood flow-based evidence for mechanisms of low- versus high-frequency transcutaneous electric acupoint stimulation analgesia: a perfusion fMRI study in humans. Neuroscience. 2014;268:180-93.

20. Lin A, Jianping D, Baixiao Z, J T, Shaoping F, Shaowu L, et al. Investigation of analgesic mechanism of acupuncture: a fMRI study(Chinese version). Chinese Journal of Medical Imaging Technology. 2004(08):1197-200.

21. Cheng Y, Lin CP, Liu HL, Hsu YY, Lim KE, Hung D, et al. Expertise modulates the perception of pain in others. Current biology : CB. 2007;17(19):1708-13.

22. Lamei L, Fajin L, Zhongjie G, Song, Hongwu X, Xi T, et al. Effect of Acupuncture Stimulation of Zusanli (ST 36) on Cerebral Regional Homogeneity in Volunteer Subjects with Different Constitutions: A Resting state fMRI Study (Chinese version). Acupuncture Research. 2013;38(4):306-13.

23. LaMei L, Xuezhi L, Fajin L, Zhongjie G, Song, Hongwu X, et al. Acupuncture analgesia effect in different sensitive constitution: a resting-state fMRI study (Chinese version) Journal of The Third Military Medicine University. 2013;35(6):547-52.

24. Lin M, Xuezhi L, Nini F, Xiaoguang Y, Xiaofang X, Fei L, et al. ASL-based Observation of Central Nervous System Responses to Acupuncture Analgesia for People with Different Sensitivities(Chinese version). Acupuncture Research. 2018;43(5):319-25.

25. Niu X, Zhang M, Liu Z, Bai L, Sun C, Wang S, et al. Interaction of acupuncture treatment and manipulation laterality modulated by the default mode network. Molecular pain. 2017;13:1744806916683684.

26. Jiliang F, Kathleen HK, Jing L, Erika N, Kehua Z, Xiaoling W, et al. Deqi and Sharp pain during acupuncture at Taichong eliciting the opposite functional brain network effects- an fMRI study. Chinese imaging journal of integrated traditional and western medicine. 2012;10(1):4-9,封2.

27. Wenjin B, Junping Z, Bo J, Yukun Z, JIE Y, Zhichang F. A preliminary study on the central analgesic mechanism of acupuncturing the stomach meridian foot-Yangming by fMRI (Chinese version). Chinese Journal Magnetic Resonance Imaging. 2020;11(11):979-84.
