# Supplementary material for: Neuroimaging in the Understanding of Acupuncture Analgesia: A Review of Acupuncture Neuroimaging Study Based on Experimental Pain Models
Source: Front Neurosci. 2021 May 20;15:648305. doi: 10.3389/fnins.2021.648305 (PMC8172961; doi:10.3389/fnins.2021.648305)
Supplement: Supplementary file 4 [file Table_3.DOCX]

**Supplementary Table 4. The overview of the 27 included studies.**

| Study  No. | Pain Model | Intervention Protocol | | | | | Scanning | Results |
| --- | --- | --- | --- | --- | --- | --- | --- | --- |
|  | **Pain**  **Model** | **Control types** | **Acupoints Selection** | **Acupuncture manipulation** | **Needle**  **Sensation** | **Pain scale** | **Scanning Techniques** |  |
| S01(1) | Heat Pain | Different intensity | Hand dorsum | TENS | Not depicted | NRS | EEG | Functional connectivity between S1/M1 and the mPFC (high intensity TENS vs low intensity TENS) |
| S02(2) | Heat Pain | VA vs SA | SP 6; SP 9 | MA | Deqi | ERS | fMRI | Insula(↑) (VA)  rACC(↓)-(watching a video of acupuncture previously) |
| S03(3) | Heat Pain | VA vs SA | LI 4 | MA | Not depicted | NRS | fMRI | Ventral striatum(Acupuncture treatment-Acupuncture stimulation); S2 and dlPFC (Acupuncture treatment) |
| S04(4) | Heat Pain | Different acupoints | SP 1; LR 1; Ren 2 | EA | Deqi | VAS | fMRI | rACC, Posterior cerebellum (↑) (Needle effect) |
| S05(5) | Heat Pain | Different intensity | SP 1; LR 1 | EA | Deqi | VAS | fMRI | More robust supraspinal sedative effect (Optimal EA-Minimal EA) |
| S06(6) | Heat Pain | VA vs SA+ Different intensity | LI 3; LI 4 | EA | Deqi | VAS | fMRI | Functional connectivity between PAG, PCC and Precuneus (VA>SA);  PAG and Anterior insula (SA<VA) |
| S07(7) | Heat Pain | VA vs SA+  VP vs SP | LI 4; SI 4 | EA | Deqi | GSAS | fMRI | Greater signal Fmri signal decreased in pain related regions insula/putamen/claustrum/superior temporal gyrus and left inferior frontal gyrus; (SA+high expectation-VA+high expectation) |
| S08(8) | Heat Pain | VP+Others | LI 4; SI 4 | EA | Deqi | GSAS | fMRI | rACC/MPFC, OPFC and dlPFC (VA+high expectation- VA+low expectation) |
| S09(9) | Heat Pain | VA vs SA | LI 4 | MA | Deqi | GSAS | fMRI +PET | OFC, Insula, and Pons (fMRI signal changes, VA - SA);  OFC, mPFC, Insula,Thalamus, and ACC (PET signal changes, VA - SA) |
| S10(10) | Heat Pain | SA+Others | Sham LI 4; Sham SI 3 | MA | Not depicted | VAS | fMRI | rACC, PFC,IPL, Supramarginal gyrus, and Anterior insula (SA activate) |
| S11(11) | Heat Pain | Different intensity | ST 36; SP 6 | EA | Not depicted | Not depicted | fMRI | Bilateral S2 and Insula, contralateral ACC and Thalamus (EAs);  contralateral S1, supplementary motor area, and ipsilateral STG(↑); bilateral Hippocampus (2-Hz EA)(↓);  contralateral IPL, ipsilateral ACC, Nucleus accumbens, and Pons(↑); contralateral Amygdala(↓) (100-Hz EA) |
| S12(12) | Electrical Pain | Post vs Pre | LI 4; LR 3; ST 36 | MA | Deqi | VAS | fMRI | S1, Insula, Cingulate gyrus and Prefrontal areas (EA) |
| S13(13) | Electrical Pain | VA vs SA | SP 6, ST 36 | EA | Not depicted | NRS | EEG | θ power near contra-lateral centro-parietal area; β activity near contra-lateral prefrontal cortex, ipsi-lateral inferior frontal and temporal lobe, and ipsi-lateral occipito-parietal cortex. |
| S14(14) | Electrical Pain | VP vs SP | LI 4 | EA | Not depicted | VAS | ERP | The amplitudes of SEP components (P170 and N280) located in Suprasylvian operculum and ACC (↓) (EA) |
| S15(15) | Electrical Pain | Others | PC 6; PC 5 | EA | Not depicted | Not depicted | ERP | Inhibit the brain evoked potentials induced by pain stimulation |
| S16(16) | Injections of Hypertonic Saline | VA vs SA | BL 40 | MA | Deqi | VAS | fMRI | Limbic system and DMN (↓); the attentional and somatosensory systems (↑) (VA-SA) |
| S17(17) | Injections of Hypertonic Saline | VA vs SA | BL 40 | MA | Deqi | VAS | fMRI | Insula(↑), ACC(↓) (VA-SA) |
| S18(18) | Cold Pain | Different intensity | ST 36; SP 6 | EA | Not depicted | VAS | fMRI | S2, mPFC, and ACC(↑); S1, parietal superior lobule, and caudal ACC (↓) (EA) |
| S19(19) | Potassium Iontophoresis Method | Different intensity | LI 4; PC 8 | TEAS | Deqi | VAS | fMRI | DMN and other cortical regions in the group(2-Hz TEAS);  Ventral striatum and dACC(100-Hz TEAS) |
| S20(20) | Potassium Iontophoresis Method | VP vs SP | LR 3 | MA | Deqi | VAS | fMRI | ACC and thalamus(↓) |
| S21(21) | Video Stimuli | Others | Body parts (mouth hands, and feet region) | MA | Not depicted | VAS | fMRI | Anterior insula, S1, PAG and ACC (Normal group activate);  mPFC, sPFC and temporoparietal junction (Expertise group activate) |
| S22(22) | Pressure Pain | Insensitive group, Normal group and Sensitive group | ST 36 | MA | Deqi | VAS | fMRI | Sensitive group:  ReHo ↑: left brainstem, bilateral cerebellum posterior lobes, left ITG, basal ganglia, the left insula lobe, ACC , frontal lobe, IPL, and the right supplementary motor area,  Reho↓: bilateral occipital lobes, fusiform gyrus , posterior central gyrus, the right posterior cingutate， the left temporal lobe and the left paracentral lobule. |
| S23(23) | Pressure Pain | Insensitive group, Normal group and Sensitive group | ST 36 | MA | Deqi | VAS | fMRI | Normal Group:  ↑cerebellum posterior lobe, brainstem, limbic lobe, inferior temporal gyrus， and right caudate;  ↓postcentral gyrus， left precentral gyrus， and bilateral occipital lobes；  Sensitive Group:  ↑cerebellum posterior lobe， fusiform gyrus，ITG, basal ganglia， frontal lobe, ACC, right supramarginal gyrus, right supplementary motor area, and left insular lobe;  ↓left middle temporal gyrus, left postcentral gyrus, posterior cingulate, and bilateral occipital lobes |
| S24(24) | Pressure Pain | Different intensity | ST 36 | MA | Deqi | VAS | ASL | ACC, mSPG (Different sensitivities on the effectiveness of acupuncture analgesia) |
| S25(25) | Capsaicin Allodynia Pain | VA vs SA | LI 4 | EA | Deqi | MGPQ | fMRI | DMN, including SPL, IPL, Precuneus, and PCC |
| S26(26) | Tactile stimulation | Acupuncture deqi; Acupuncture pain; Tactile stimulation | LV 3 | MA | Deqi | Not depicted | fMRI | Deqi without sharp pain: the limbic‐paralimbic‐neocortical network↓ |
| S27(27) | Stabbing pain | Pain stimulation→acupuncture stimulation | ST 36, ST 37 | EA | Deqi | Not depicted | fMRI | Frontal and temporal cortex, the limbic system, the thalamus, the cerebellum activated |

**Abbr.**

VA. Verum acupuncture; SA. Sham acupuncture; VP. Verum acupoints; SA. Sham acupoints;

VAS. Visual Analogical Scale; GSAS. Gracely Sensory and Affective Scales; NRS. Numerical Rating Scale; ERS. Expectations for Relief Scale; MPQ. McGill Pain Questionnaire.

fMRI. functional Magnetic Resonance Imaging; ERP. Event-Related Potentials; PET. Positron Emission Tomography; ASL. Arterial Spin Labeling

OFC. Orbitofrontal Cortex; dOPFC. dorsal Oribital Prefrontal Cortex; ACC. Anterior Cingulate Cortex; MCC. Medial Cingulate Cortex; PCC. Posterior Cingulate Cortex; rACC. rostral Anterior Cingulate Cortex; PFC. Prefrontal Cortex; mPFC. Medial Prefrontal Cortex; lPFC. lateral Prefrontal Cortex; dlPFC. dorsolateral Prefrontal Cortex; IPL. Inferior Parietal Lobule; MCC. Medial Cingulate Cortex; IFL. Inferior Frontal Lobe; PAG. Periaqueductal Gray; S2. Secondary Somatosensory Cortex; S1/M1. Primary Somatosensory Cortex; STG. Superior Temporal Gyrus; SPL. Superior Parietal Lobule; mSPG. medial Superior Frontal Gyrus; SPL. Superior Parietal Lobule; DMN. Default Mode Network; ITG. Inferior Temporal Gyrus

**Reference**

1. Peng WW, Tang ZY, Zhang FR, Li H, Kong YZ, Iannetti GD, et al. Neurobiological mechanisms of TENS-induced analgesia. Neuroimage. 2019;195:396-408.

2. Cao J, Tu Y, Orr SP, Lang C, Park J, Vangel M, et al. Analgesic Effects Evoked by Real and Imagined Acupuncture: A Neuroimaging Study. Cereb Cortex. 2019;29(8):3220-31.

3. Lee IS, Wallraven C, Kong J, Chang DS, Lee H, Park HJ, et al. When pain is not only pain: inserting needles into the body evokes distinct reward-related brain responses in the context of a treatment. Physiology & behavior. 2015;140:148‐55.

4. Leung A, Zhao Y, Shukla S. The effect of acupuncture needle combination on central pain processing--an fMRI study. Molecular pain. 2014;10:23.

5. Shukla S, Torossian A, Duann JR, Leung A. The analgesic effect of electroacupuncture on acute thermal pain perception--a central neural correlate study with fMRI. Molecular pain. 2011;7:45.

6. Zyloney CE, Jensen K, Polich G, Loiotile RE, Cheetham A, LaViolette PS, et al. Imaging the functional connectivity of the Periaqueductal Gray during genuine and sham electroacupuncture treatment. Molecular pain. 2010;6:80.

7. Kong J, Kaptchuk TJ, Polich G, Kirsch I, Vangel M, Zyloney C, et al. Expectancy and treatment interactions: a dissociation between acupuncture analgesia and expectancy evoked placebo analgesia. Neuroimage. 2009;45(3):940-9.

8. Kong J, Kaptchuk TJ, Polich G, Kirsch I, Vangel M, Zyloney C, et al. An fMRI study on the interaction and dissociation between expectation of pain relief and acupuncture treatment. Neuroimage. 2009;47(3):1066‐76.

9. Dougherty DD, Kong J, Webb M, Bonab AA, Fischman AJ, Gollub RL. A combined [11C]diprenorphine PET study and fMRI study of acupuncture analgesia. Behavioural brain research. 2008;193(1):63-8.

10. Kong J, Gollub RL, Rosman IS, Webb JM, Vangel MG, Kirsch I, et al. Brain activity associated with expectancy-enhanced placebo analgesia as measured by functional magnetic resonance imaging. The Journal of neuroscience : the official journal of the Society for Neuroscience. 2006;26(2):381-8.

11. Zhang WT, Jin Z, Cui GH, Zhang KL, Zhang L, Zeng YW, et al. Relations between brain network activation and analgesic effect induced by low vs. high frequency electrical acupoint stimulation in different subjects: a functional magnetic resonance imaging study. Brain research. 2003;982(2):168-78.

12. Theysohn N, Choi KE, Gizewski ER, Wen M, Rampp T, Gasser T, et al. Acupuncture-related modulation of pain-associated brain networks during electrical pain stimulation: a functional magnetic resonance imaging study. Journal of alternative and complementary medicine (New York, NY). 2014;20(12):893-900.

13. Weiting Z, Fei L, Yingwei Q, Ying W, Jingyu Z, J.WOODWORD D, et al. Modulation of pain signal processing by electric acupoint stimulation: an electroencephalogram study (Chinese version). Journal of Peking University. 2003;35(3):236-40.

14. Zeng Y, Liang XC, Dai JP, Wang Y, Yang ZL, Li M, et al. Electroacupuncture modulates cortical activities evoked by noxious somatosensory stimulations in human. Brain research. 2006;1097(1):90-100.

15. Beijing RGoAAi. Event-related potential evoked by suggestive sensitivity and acupuncture on pain stimuli(Chinese version). Journal of Psychology. 1980(2).

16. Shi Y, Liu Z, Zhang S, Li Q, Guo S, Yang J, et al. Brain Network Response to Acupuncture Stimuli in Experimental Acute Low Back Pain: An fMRI Study. Evidence-based Complementary and Alternative Medicine. 2015;2015.

17. Ziping L, Wen W, Shanshan Z, Shigui G, Jianming Y. Pain matrix response to acupuncture stimuli in individuals with acute Low back pain: an fmri study (Chinese version). Chinese Journal of Pain Medicine. 2013;19(4):201-5,.

18. Zhang WT, Jin Z, Huang J, Zhang L, Zeng YW, Luo F, et al. Modulation of cold pain in human brain by electric acupoint stimulation: evidence from fMRI. Neuroreport. 2003;14(12):1591-6.

19. Jiang Y, Liu J, Liu J, Han J, Wang X, Cui C. Cerebral blood flow-based evidence for mechanisms of low- versus high-frequency transcutaneous electric acupoint stimulation analgesia: a perfusion fMRI study in humans. Neuroscience. 2014;268:180-93.

20. Lin A, Jianping D, Baixiao Z, J T, Shaoping F, Shaowu L, et al. Investigation of analgesic mechanism of acupuncture: a fMRI study(Chinese version). Chinese Journal of Medical Imaging Technology. 2004(08):1197-200.

21. Cheng Y, Lin CP, Liu HL, Hsu YY, Lim KE, Hung D, et al. Expertise modulates the perception of pain in others. Current biology : CB. 2007;17(19):1708-13.

22. Lamei L, Fajin L, Zhongjie G, Song, Hongwu X, Xi T, et al. Effect of Acupuncture Stimulation of Zusanli (ST 36) on Cerebral Regional Homogeneity in Volunteer Subjects with Different Constitutions: A Resting state fMRI Study (Chinese version). Acupuncture Research. 2013;38(4):306-13.

23. LaMei L, Xuezhi L, Fajin L, Zhongjie G, Song, Hongwu X, et al. Acupuncture analgesia effect in different sensitive constitution: a resting-state fMRI study (Chinese version) Journal of The Third Military Medicine University. 2013;35(6):547-52.

24. Lin M, Xuezhi L, Nini F, Xiaoguang Y, Xiaofang X, Fei L, et al. ASL-based Observation of Central Nervous System Responses to Acupuncture Analgesia for People with Different Sensitivities(Chinese version). Acupuncture Research. 2018;43(5):319-25.

25. Niu X, Zhang M, Liu Z, Bai L, Sun C, Wang S, et al. Interaction of acupuncture treatment and manipulation laterality modulated by the default mode network. Molecular pain. 2017;13:1744806916683684.

26. Jiliang F, Kathleen HK, Jing L, Erika N, Kehua Z, Xiaoling W, et al. Deqi and Sharp pain during acupuncture at Taichong eliciting the opposite functional brain network effects- an fMRI study. Chinese imaging journal of integrated traditional and western medicine. 2012;10(1):4-9,封2.

27. Wenjin B, Junping Z, Bo J, Yukun Z, JIE Y, Zhichang F. A preliminary study on the central analgesic mechanism of acupuncturing the stomach meridian foot-Yangming by fMRI (Chinese version). Chinese Journal Magnetic Resonance Imaging. 2020;11(11):979-84.
